# Enhancing Pediatric Palliative Care for Latino Children and Their Families: A Review of the Literature and Recommendations for Research and Practice in the United States

**DOI:** 10.3390/children5010002

**Published:** 2017-12-22

**Authors:** Sara Muñoz-Blanco, Jessica C. Raisanen, Pamela K. Donohue, Renee D. Boss

**Affiliations:** 1 Department of Pediatrics, Johns Hopkins University School of Medicine, Baltimore, MD 21205, USA; smunozb1@jhmi.edu (S.M.-B.); pdonohue@jhmi.edu (P.K.D.); 2 Clinical Ethics, Johns Hopkins Berman Institute of Bioethics, Baltimore, MD 21205, USA; jraisan1@jhu.edu; 3 Department of Population, Family and Reproductive Health, Johns Hopkins Bloomberg School of Public Health, Baltimore, MD 21205, USA

**Keywords:** pediatric palliative care, Latino health, chronic illness, children with medical complexity

## Abstract

As the demand for pediatric palliative care (PC) increases, data suggest that Latino children are less likely to receive services than non-Latino children. Evidence on how to best provide PC to Latino children is sparse. We conducted a narrative review of literature related to PC for Latino children and their families in the United States. In the United States, Latinos face multiple barriers that affect their receipt of PC, including poverty, lack of access to health insurance, language barriers, discrimination, and cultural differences. Pediatric PC research and clinical initiatives that target the needs of Latino families are sparse, underfunded, but essential. Education of providers on Latino cultural values is necessary. Additionally, advocacy efforts with a focus on equitable care and policy reform are essential to improving the health of this vulnerable population.

## 1. Introduction

Medical advancements and technology have decreased the number of deaths for medically fragile children over time [1,2,3]. With the increased life expectancy of this population, the demand for pediatric palliative care (PC) and end-of-life (EOL) care is also on the rise. In the United States, pediatric PC services are rapidly developing with the goal of improving quality of life for children with serious illness and their families [4]. However, research exposes disparities in pediatric PC use in the US by race/ethnicity such that European Americans are more likely to use pediatric PC services than Latinos living in the US [5]. It has also been shown that Latino children incur lower expenditures in hospice care than non-Latino children suggesting that there may be delays in admission to care or persistent barriers to pediatric PC services for Latino families in the US [6]. For the purposes of this paper, we use the term “Latino” to represent people of all genders that emigrated to the United States from Latin America and/or have strong cultural ties to this region. Individuals within this population might use other labels such as Latino/a, Latina, Latinx, Latin American, and Hispanic to identify their ethnicity. Furthermore, data suggest that minority pediatric cancer patients receive more intensive care at the EOL and are more likely to die in the hospital [7]. This is especially concerning given the growing body of work suggesting that early integration of pediatric PC services results in improved outcomes [8,9]. The objective of this paper is to review existing theory and research relating to PC services for Latino children and their families. We analyze the findings within current cultural and political contexts and present recommendations for future clinical care, research, and policy related to pediatric PC for Latino populations in the US.

## 2. Methods

We searched MEDLINE and Pubmed from inception to August 2017 for English language articles relevant to palliative care in the Latino population, particularly of pediatric patients using the terms: palliative care, Latino, Hispanic, pediatric palliative care. We then reviewed bibliographies of relevant studies to broaden our search. At a later stage, we also searched for references related to the current political context and its effect on the Latino US population. Two authors made the final selections and critically reviewed the existing data and models of care to identify current state of pediatric palliative care services for Latino children in the US.

## 3. Review of the Literature

### 3.1. Cultural Considerations Relevant to Palliative Care for Latino Pediatric Populations

Latino individuals make up the largest ethnic minority group in the US, and it is estimated that the number of Latino people will reach 28.6% of the total US population by the year 2060 [10]. People of Latino ethnicity are often treated as one monolithic group when interacting with health care systems and participating in research, but this perception of Latinos as homogeneous is flawed; many Latinos that reside in the US have roots in different countries and have different cultural backgrounds. Therefore, clinicians providing care to Latino patients in the US are likely to encounter an array of beliefs, values, and languages. However, shared experiences among Latinos, such as Spanish colonization of indigenous people in Latin America and the Caribbean [11], language [11,12], and ethnic discrimination [12] contribute to a sense of unity and community. These experiences foster cultural connections in ways that influence medical care. Here, we review common Latino cultural values surrounding family, gender roles, religion, spirituality, and decision-making preferences that may influence the availability, acceptability, and receipt of pediatric PC services within this ethnic group.

The role of the extended Latino family in making decisions for children with serious illness may be of particular importance. *Familismo*, a value of commitment and loyalty to immediate and extended family [11], is important for many Latinos [13,14,15,16,17,18,19,20]. Latino families are often tight-knit and large, which can lead to increased support and pooling of resources [16]. However, it can also complicate discussions around care, as it may lead to high levels of family involvement in decision-making [17]. It may be difficult to arrange large family meetings or reach consensus and understanding of the dying process [17], especially if there are language barriers between families and providers. This additional consultation with extended family may lead to delays in care [20,21].

Cultural perceptions of masculinity and femininity within the Latino community, often referred to as *machismo* and *marianismo/hembrismo*, may also shape care and decision-making for children with serious illness. Specifically, gender roles influence health-seeking behavior, medical decision-making, and caretaking such that mothers are often expected to act as caretakers and facilitators of health-seeking behavior. In contrast, fathers are often conditioned not to engage in daily caretaking for children, yet they are expected to lead decision-making surrounding health care, while mothers may feel obligated to obtain the father’s permission to participate [14,16]. Gender norms within Latino populations may contribute to the reasons why some male family members deny engagement with PC services, because sharing feelings or concerns about illness may not be acceptable to some Latino men; they may prefer that their female family members be the focus of any support services and efforts [13].

While spirituality, religious community, and belief systems may be a source of support for families and children [22,23], it has also been suggested that spirituality and/or religiosity can create a barrier to care for some Latino families [17,24]. For example, strong religious beliefs, such as belief in miracles, may lead families to deny the dying process [13,17] or to view pain and suffering as a test of faith [24,25]. Religious values around the sanctity of human life and life as a gift from God may also influence EOL care in that for some Latinos, brain death may not be sufficient to justify withdrawal of life support [26]. Finally, *fatalismo*, or a belief in fate that one’s future is predetermined, may also play a role in deterring Latinos from seeking care [14,16]. Abraído-Lanza et al. [27] posit that inaccessibility and discrimination within the healthcare system may reinforce feelings about *fatalismo* in Latino populations because of the lack of access to adequate and appropriate services. Structural barriers like racial bias, poverty, health insurance inaccessibility, and immigration barriers may reaffirm fatalistic ideology among people that experience oppression, undermining access to care even further [27]. These beliefs may influence some Latinos to postpone or refuse pediatric PC services. Multiple studies demonstrate that health care providers rarely engage patients in discussions of how religion/spirituality impact their health and health decisions [28,29]; this failure may have added significance for Latino families where reasons for delaying or refusing care may be misunderstood and inadequately addressed.

*Respeto* refers to the way Latinos interact with each other according to a person’s age, gender, socioeconomic status, and authority [15,21]. This can be especially influential to the parent-physician relationship. For example, deference to authority figures may hinder families from questioning recommendations given by healthcare providers, even when those recommendations are incongruent with families’ wishes, beliefs, and cultural norms [15,21,24]. Unfortunately, this can lead to misunderstandings and non-compliance with care [15]. In turn, providers are expected to behave according to Latino hierarchical norms by showing *respeto* and including extended family members in decision-making [15,21,24].

Because PC providers commonly engage with parents of children with serious illness in making medical decisions, it is also important to be aware of potential cultural variability in preferred decision-making roles. It is common for clinicians to assume that Latino patients prefer passive roles in medical decision-making [18,30,31,32]. However, there is little empirical data supporting this passive role preference. On the contrary, research suggests that people of Latino ethnicity living in the US may prefer more active or shared decision-making roles [21,33], especially when compared to Latinos living in Latin America [33]. Regardless of decisional-control preferences, Latino patients report that they want to know their diagnosis and prognosis [34]. Clinicians in the US are tasked with developing an understanding of how cultural contexts influence individual preferences for care within this heterogeneous population.

**Figure 1:**
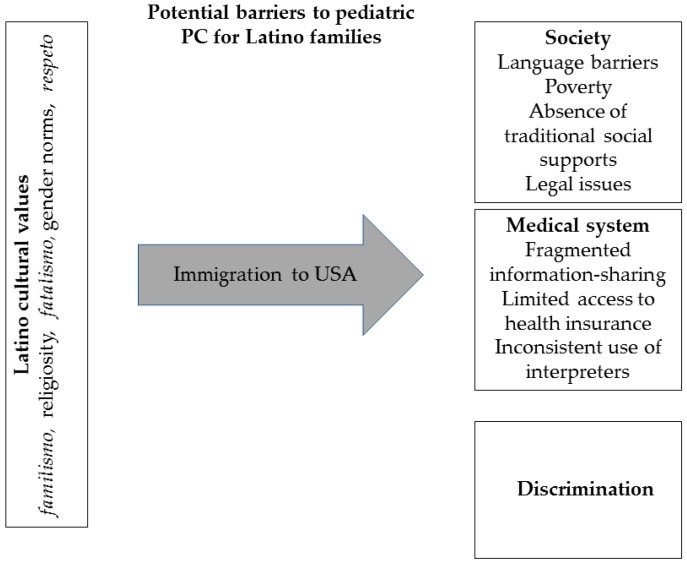
Mediators of the pediatric palliative care (PC) experience for latino families.

This previous research on the influence of cultural beliefs on health-seeking behavior lays a foundation for contextualizing the inequity observed in pediatric PC of Latino populations (Figure 1).

### 3.2. Insights from Latin America

Overall, PC is underdeveloped in Latin America [35,36,37]. Because of this, Latino patients in the US may have a limited understanding of the role of PC. However, PC services vary greatly from region to region in Latin America [35,38]. Therefore, understanding and acceptance of pediatric PC by Latino families in the US may also depend on where the family’s roots are situated. Literature on pediatric PC in Latin America could inform care of Latino children in the US. However, research on best practices in PC is sparse and underfunded in Latin America [35,39,40], and while children and their families have unique PC needs, Latin American literature does not explicitly discuss considerations for PC in pediatric populations. International collaboration may be helpful to advance the research agenda in this topic and inform US practice. In the meantime, clinicians are encouraged to inquire families’ understanding of and prior experience with pediatric PC.

### 3.3. Pediatric Palliative Care Experiences of Latino Families in the US

Over the past decade, literature focusing on PC in the US Latino population, which is not robust, has expanded to include the adult and pediatric PC and hospice experience, barriers to quality care, and suggestions for clinical care and research. There remain substantial gaps in the evidence base regarding PC for Latino children. In this section, we will review what is known about PC delivery in the US for Latino children and their families.

Poverty, absence of traditional social supports, and difficulties with the healthcare system are important mediators of PC experiences for Latino families [41]. Poverty can interfere with cultural practices around death and the bereavement process, as when some families are forced to opt for cremation [41]. Furthermore, the high cost of healthcare is an added source of burden for poor families [19,42]. In addition, geographic separation from family and traditional social supports can lead to feelings of isolation, as expected given the cultural value of *familismo* [16,41,43]. Despite this, however, Latino families also report a sense of trade-off that being in the US allows their child to receive the best care possible [41]. Lastly, some families also struggle with the complexity of the US healthcare system. For example, having multiple doctors, an experience different than in many countries in Latin America, can lead to fragmented information-sharing and thus affect communication and trust [41,44].

The importance of honest communication, direct communication by medical providers, emotional, financial, and physical strains associated with caregiving, and anxieties felt around time of the child’s death are important mediators of the pediatric hospice experience [43]. Latino families, in particular, value the opportunity that hospice provides for their loved one to be home [42,43]. In addition, compared to English-speaking counterparts, Spanish-speaking families are less distressed by issues of symptom management at the end of life, report difficulties with doing bedside nursing care [43], and express desire to reduce caregiver burden [19], suggesting differences in the hospice experience between Latinos and other racial/ethnic groups.

Limited English proficiency is a barrier to quality pediatric PC [41,43]. Most Latino immigrants speak Spanish and only a fourth report fluency in English [45]. However, most medical care in the US is delivered in English. While there are health care providers in the US that report being fluent in Spanish, a majority of these providers are non-Latino and are not native Spanish speakers. Thus, even for these providers, communication may still be difficult [46]. The meaning of the word hospice, for example, is not easily translated into Spanish and patients often think that it refers to a place and not a service [17].

Language barriers, which often lead to miscommunication, can be a source of mistrust between Latino families and PC providers [19,44]. An uncertain understanding of their child’s condition can leave these families feeling isolated, confused, and distrustful of the healthcare system [44]. Due to the inability to speak English, some families fear not being able to do what is needed to care for their child [43], others report difficulty with reaching out for help [41]. Some parents also report dissatisfaction with communication; for example, when interpreter-facilitated family meetings are held in the child’s room and parents and children hear difficult news at the same time [43]. Furthermore, parents with limited English proficiency report feeling distressed when they do not understand the implications of the information they receive, which leads to dissatisfaction with care and long-term psychosocial stress [47]. This can be especially detrimental when discussing EOL issues.

Discrimination based on race/ethnicity remains a problem in US health care systems across a broad range of care sites and is more difficult to address than the language barriers [19,41,48]. Discrimination has also been reported in the delivery of PC services. Davies et al reported that Mexican-American families felt discriminated against based on race, language, socio-economic status, and appearance [48]. They also reported feeling confused, angry, and hurt by these experiences. However, few families spoke up about their concerns with care; most assumed a passive/submissive role, as dictated by cultural norms of *respeto* of authority figures, or for fear of recrimination against their child [48].

Documented disparities in rates of advance directives for pediatric Latino patients are sparse. However, research among adult patients may have implications for families of children with serious illness. In a study of older Latino adults, 84% reported that they would want comfort-focused care if seriously ill, a proportion that contradicts the high rates of aggressive treatments at the EOL observed in this population. However, over three quarters of these participants reported that they did not have an advanced care directive and almost half had not discussed their preferences with their family or doctor [18]. Kelley et al., argue that these missed opportunities put patients at risk of receiving aggressive and unwanted treatment at the EOL [18]. This could also be true for children. A study in which a majority of the participants were Latino parents found that 62% had never heard of advance directives and that 82% had never discussed advance directives. However, after being educated, 49% of parents expressed interest in creating an advance directive for their chronically ill child. Of note, Spanish-speaking Latino participants were less likely to have knowledge on advance directives than English-speaking participants [49]. Though there are global difficulties with expanding advance directives overall, health system policies which work to address these may still miss those patient populations—like Latino patients—that experience discrimination within the health care system or have difficulty communicating their desires with providers.

Barriers in access to health insurance for Latino children have historically presented issues for care [50,51,52,53]. Additionally, chronically ill children that are Latino have the lowest rates of insurance coverage when compared to chronically ill children of other ethnic or racial groups in the US [52,54]. While health care reform in the US, namely the Affordable Care Act (ACA), increased insurance rates across the board, Latinos still have the highest probability of being uninsured compared to White and African American populations [55]. Additionally, the recent challenges to the ACA, such as the Senate Tax Cuts and Jobs Act, may reverse initiatives directed at equity in health insurance access. These policy changes have implications for medically complex Latino children in that they may be underinsured, leading to decreased access to pediatric PC services.

Poverty and political turmoil have led many people from Latin America to migrate to the US or other areas of the world [21], and the vast majority of undocumented immigrants residing in the US are from Latin America [56]. These undocumented immigrants with life-limiting illness face additional barriers to care such as fear of deportation, lack of insurance, and limited access to services, including PC and hospice. While some children who are undocumented are able to access medical coverage due to state-based policies in a small number of states (i.e., California, New York, Illinois, Massachusetts, Washington, and the District of Columbia), in a majority of states, undocumented immigrants are unable to access health insurance through Medicaid, Medicare, or the Insurance Marketplace established by the ACA [57]. These restrictions contribute to many Latino people being denied access to necessary medical care, which some consider to be a violation of their human rights. Jaramillo and Hui describe the difficult experience of an undocumented young adult immigrant with advanced cancer at the EOL [42]. Language and cultural barriers, delayed diagnosis, limited social support, increased financial burden, limited access to EOL care, and fear of deportation are just a handful of issues that undocumented immigrants face that may impede care [42]; undocumented children with chronic illness and their families face these hardships as well. US anti-immigration legislation poses a threat to the health and well-being of many Latinos, including medically complex children and their families. For example, elimination of the Deferred Action for Childhood Arrivals (DACA) Program will leave some Latinos unable to work or continue their education in the US and at risk of deportation, which will likely increase poverty among this population, decrease educational attainment and employer-based health insurance coverage, and contribute to increasing inequity in access to pediatric PC services [52]. To our knowledge there are no studies that focus directly on the pediatric PC experience for immigrant, uninsured children in the US.

As illustrated in this review, the literature on the pediatric PC experience for Latino children and their families is sparse. The growing body of evidence on the delivery, experience, and gaps in PC drawn from the adult literature can serve as a starting point and guide for pediatric PC providers and researchers who serve Latino families. Absence of traditional social supports, language barriers, difficulties with navigating and understanding the US healthcare system, and discrimination are important mediators of the pediatric PC experience of Latino families, though this is likely just the tip of the iceberg.

## 4. Discussion

### 4.1. Improving Clinical Care

Latinos are often thought of and treated as one group in the US; however, the heterogeneity of this population cannot be ignored. Even though language is a common bond between Latino populations, culture, beliefs, and attitudes that shape their experience and understanding of pediatric PC may differ by country of origin.

#### 4.1.1. Becoming Patient Advocates

Vulnerability of Latino patients framed by language barriers, access to healthcare, socioeconomic hardship, and cultural differences is accentuated in serious illness and at the EOL. Thus, providers are encouraged to become avid patient advocates when caring for Latino children. Close attention to socioeconomic particularities of each family is recommended. This includes being aware of competing agendas between hospital administration and patient discharge planning and outpatient care, increasingly important for those patients who are uninsured [58]. In addition, proactively but empathetically assessing the economic situation and home environment of a family can help determine the kind of care the family can provide at home [41]. This is particularly important when discussing medical technology with families, for example.

#### 4.1.2. Bridging the Language Barrier

Language barriers have long been recognized as a limitation to receiving quality care. It is also important to recognize that language fluency does not equal cultural competency. Thus, it is best to use simple and clear language when discussing terminal illness issues and avoid using euphemisms that are likely to be lost in translation [24]. In addition, not using interpreters consistently has been perceived by parents as discriminatory [48], a cause of poor information-sharing, and lack of acknowledgement regarding their emotions and concerns [47]. Increasing access to interpretation services and increasing the number of native Spanish-speaking staff and providers will assist in bridging the communication gaps. Even among providers that are highly proficient and fluent in Spanish, it may still be best to use an interpreter. Children and relatives should not be used as interpreters, especially when discussing EOL care and decision making due to the sensitive nature of the conversation and confidentiality issues.

#### 4.1.3. Cultural Humility

Cultural humility centered around common Latino cultural values and strategies on how to overcome language barriers should be a priority to everyone who provides pediatric PC to Latino children [15,47]. Employing universal strategies for communication (i.e., ask-tell-ask model or teach-back method); assessing acculturation with open-ended, respectful questions; and, practicing strategies for establishing trust (i.e., naming the emotion or asking about discrimination experiences) are also encouraged [16]. Latino cultural values need to be recognized, taught, and integrated into a culture-centered model of PC [15]. This model is a framework in which to weigh the influence of acculturation and ethnic identity on a Latino patient’s and family’s experience of PC. However, one must be conscious that given the variability in decisional-control and information-sharing preferences among Latinos, individual assessment of each patient and family is equally important.

#### 4.1.4. Continuity of Care

The stratification of care characteristic of the US healthcare system can be confusing for some Latino families since in many Latin American countries, one physician is in charge of directing care and communicating with the family. As such, Latino families may not be familiar with a multi-disciplinary approach to care. Assigning a continuity provider, preferably one who speaks Spanish and has established rapport with the family, may help with trust building and comprehension of information [41,44].

### 4.2. Implications for Research

As highlighted in this narrative review, data on how to best provide quality pediatric PC to Latino children are scarce. We found two studies that focused on the experiences of Mexican American families with pediatric PC [41] and perceived discrimination during interaction with pediatric healthcare providers [48]. While these are pioneer studies, they have limited generalizability. One other study explored English- and Spanish-speaking families’ perceptions of pediatric hospice [43]. Although exploratory in nature, and limited by a small sample size, the study provides insight on how language, and likely culture, can shape families’ experience with hospice. Language is often the first perceived barrier to care. Thus, studying the barriers to consistent use of interpreters may help decrease communication gaps.

Palliative care services in Latin America vary by region. As such, Latino families’ experience with PC can vary widely. Thus, educating Latino communities on PC, and developing evidence-based ways to do so, should be a primary goal of research. Community outreach programs [59] and media utilization, such as videos [60], specifically designed for pediatric PC are potential interventions to be studied. Likewise, culturally-competent patient navigators that address education and patient activation through home visits could serve to increase baseline knowledge of pediatric PC and facilitate care delivery [61].

Latino families often have socioeconomic realities that are different from those of white or black families [17,53,55,62]; these socioeconomic characteristics contribute to health inequity. Correspondingly, studies that explore Latino caregiver burden and search for ways in which allocation of pediatric PC resources can help ameliorate burden are also needed [43]. Lastly, more population-based studies are needed to explore potential causes for disparities in intensive care at the EOL for minority pediatric patients like those observed by Johnston et al. [7]. In particular, it will be important to elucidate if these disparities are due to healthcare system issues (i.e., access to PC) or to family preference/goals [7,63].

Pediatric PC research is limited in Latin America; financial and educational barriers hinder its development. Given the heterogeneity of the US immigrant Latino population, collaboration between US and Latin American researchers is imperative for the advancement of pediatric PC for Latino children. Limited resources and minimal expertise and training in research [37,40] are some reasons to advocate for international collaboration with developed countries. In addition, translating published literature to Spanish may help with dissemination of evidence in Latin America.

### 4.3. Policy, Advocacy and Education

Vulnerable populations, such as Latino children, depend on advocacy efforts from those who are passionate, as much as those who are in power. Advocacy can occur at many levels: individual patient, single institution, community, state, and federal. Current need is at all levels and providers are encouraged to take action.

Advocating for immigration reform that supports legal pathways to immigration and health care reform that expands health insurance coverage is important to improving access to PC for medically complex Latino children and decreasing inequity. While a small number of states have assisted undocumented immigrants in accessing health insurance, we are unaware of protections or allocation of funds to assist this population at the federal level.

Unfortunately, no official legislation exists to support parents in making advance medical directives. In the United States, at least one state, Maryland, mandates consideration of advance directives for hospitalized children through use of Medical Orders for Life-Sustaining Treatment (MOLST) [64]. This document, however, is not pediatric specific. Pediatric advanced care planning programs, nonetheless, can be successfully implemented [65]. In addition, data shows parents favor a MOLST for hospitalized children, though providers as well as parents recognize conversations about pediatric advance care planning are challenging and require good communication skills [66,67]. Communication training for providers and pediatric advance directive education for parents can help de-stigmatize conversations around advance care planning [65,66]. Furthermore, state and federal legislation is needed to implement a structured system to address pediatric advance directives [49]. Families whose children have chronic medical conditions or life-limiting illnesses would likely benefit from such a system.

Action can feel like a daunting task, and avidly advocating for patients on a day-to-day basis can lead to feelings of frustration and burnout among providers who lack necessary skills for advocacy [58]. However, the clinician’s role has inherent potential for advocacy that could be utilized. One way that this might be able to take place is through encouraging partnerships between hospitals, medical schools, community hospices, home care agencies, and Latino community resources, such as outreach programs, volunteers, or churches [41,60]. These partnerships may increase educational opportunities for providers and elicit organizational-level changes to assist with provision of care to Latino children.

**Figure 2:**
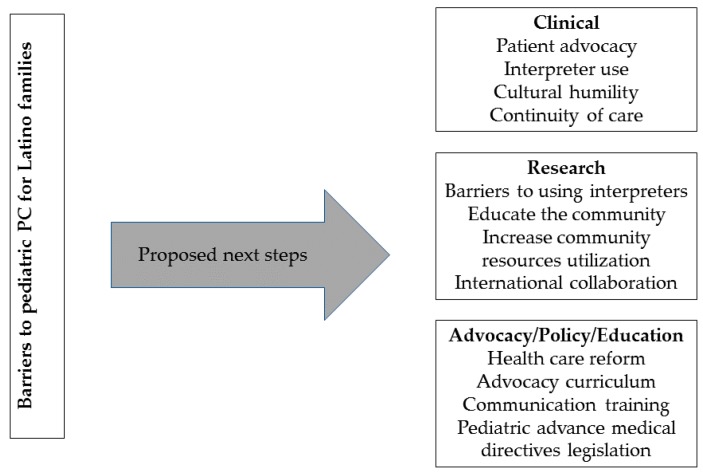
Proposed next steps to enhance the pediatric palliative care experience for Latino families.

Without doubt, change in clinical practice and more research are needed to determine the best way to deliver pediatric PC to Latino children (Figure 2). With more data, advocacy at the state and federal levels is likely to be more effective.

## 5. Conclusions

While the demand for pediatric PC is increasing with medical advancements, care is not universally available. Moreover, political, economic, and social injustices can systematically undermine access to care for Latino populations. Additionally, cultural values, clinical experiences with discrimination, and health-related policy influence Latinos’ access to care. Integrating Latino cultural values into clinical practice is key to the delivery of culturally sensitive pediatric PC for Latino children. Research focusing on provision of pediatric PC services to this population is essential in order to move forward and improve the quality of life of Latino children with serious illness and their families. Partnerships between lower-resourced countries in Latin America and higher-resourced entities, such as academic institutions in the US, would likely benefit the research agenda and lead to improvements in care for this vulnerable population. Finally, advocacy will play a large role in enhancing the availability of pediatric PC for Latino children and their families.
